# The supercooling point depression is the leading cold tolerance strategy for the variegated ladybug, [*Hippodamia variegata* (Goezel)]

**DOI:** 10.3389/fphys.2023.1323701

**Published:** 2023-12-21

**Authors:** Mahsa Khabir, Hamzeh Izadi, Kamran Mahdian

**Affiliations:** Department of Plant Protection, Faculty of Agriculture, Vali-e-Asr University of Rafsanjan, Rafsanjan, Iran

**Keywords:** cold tolerance, supercooling point, *Hippodamia variegata*, cryoprotectant, lower lethal limit

## Abstract

The variegated ladybug, Hippodamia variegata is one of the most effective predators of various pests that hibernate as adult beetles. During the overwintering period from April 2021 to March 2022, we examined the supercooling point (SCP), cold tolerance, and physiological adaptations of beetles in Kerman, Iran. The beetles exhibited their greatest cold tolerance (63.4% after 24 h at −5°C) when their SCP was lowest (−23.2°C). Conversely, from April to October 2021, the SCP reached its peak (approximately −13.0°C), while cold tolerance was at its lowest level (6.7% after 24 h at −5°C). Cryoprotectant content (trehalose, glycerol, and glucose) was at its highest level in September (11.15, 10.82, and 6.31 mg/g, respectively). The critical thermal minimum (CTmin) reached its lowest point of −2.2°C in January and February. The lowest point of the lower lethal temperature (LLT) coincided with the lowest level of the SCP and the highest level of cold tolerance (in February, LT50 = −5.3°C, SCP = −23.2°C, and survival = 77.78% at −4°C/24 h). Chill-coma recovery time (CCRT) was examined at five different temperatures and two different exposure durations. The CCRT increased with a decrease in exposure temperature and time (68.0 s at −2°C after 2 h and 102.0 s at −2°C after 4 h). As the majority of the overwintering beetle’s mortality occurred at temperatures significantly higher than SCP, the adults of H. variegata are chill-susceptible insects that primarily rely on a depressed supercooling point to cope with unfavorable conditions during the overwintering period.

## 1 Introduction

Ladybird beetles have garnered significant attention as predators of macroinvertebrates and soft-bodied insects. These predators have been extensively employed as biological control agents globally (Sailer and Debach, 1975; Obrycki and Kring, 1998; Michaud, 2012). *Hippodamia variegata* (Goezel) (Col.: Coccinellidae) is one of the most significant natural predators of the common pistachio psylla, *Agonoscena pistaciae* (Hem: Psyllidae). This ladybird beetle is commonly found in pistachio-growing regions of Iran. Both larvae and adults play a crucial role in the natural control of the common pistachio psylla, as they are predators of different developmental stages of the pest (Mehrnejad, 2003).

Every aspect of the lives of ectotherms, such as insects, including their function, behavior, distribution, and adaptability, is greatly influenced by the surrounding temperature. It is essential to comprehend how the life history of ectotherms alters to adapt to unfavorable environmental temperatures (Clarke, 1993; Sun et al., 2022). The impact of ambient temperature on insect life history is influenced by multiple factors, including species, adaptation abilities, and geographical distribution. Nonetheless, in the majority of insect species, lower temperatures generally lead to a decrease in the rate of development, resulting in longer developmental times for each stage (Ju et al., 2011). From a biological perspective, a low temperature hinders the activity of a particular species. This threshold differs across species and depends on their physiological condition (Lee, 1991; Denlinger and Lee, 2010).

*Hippodamia variegata* overwinters as adults under host plant bark (Kontodimas and Stathas, 2005). A cold winter with subzero temperatures is necessary for pistachio trees to complete their dormancy (Rahemi and Pakkish, 2009). However, these harsh conditions are detrimental to the survival and reproductive capabilities of the beetles, causing sublethal effects on their growth, development, and distribution. To survive in such extreme environmental conditions, the insects use various strategies including migration, cold tolerance, and diapause.

The life history of insects, being poikilothermic creatures, is greatly impacted by temperature. The survival, abundance, distribution, and even metabolic rate, as well as the subsequent activity, of an insect population are highly influenced by the surrounding temperature (Scaccini et al., 2020). Furthermore, the fitness and ability of animals to withstand harsh environmental conditions greatly affect their survival and lifespan. Cold acclimation, which involves gradually exposing animals to decreasing low temperatures, is an innate ability that allows them to tolerate sublethal thermal conditions by adjusting their physiological mechanisms (Terblanche et al., 2007; Clark and Worland, 2008; Enriquez et al., 2018; Enriquez and Colinet, 2019).

Diapause is a crucial adaptation strategy. It allows many insect species to suspend their development, thereby enabling them to survive in unfavorable environmental conditions. Additionally, diapause helps synchronize the life cycles of individuals and maintain the population (Gill et al., 2017; Tougeron, 2019). Diapause alone cannot guarantee survival. In addition to diapause initiation, cold hardiness is also necessary to ensure the insect’s life. Cold hardiness, also known as cold tolerance, refers to an insect species’ ability to prevent cold injuries and survive exposure to harsh winter conditions (Andreadis and Athanassiou, 2017; Pourani et al., 2019). In most insects, there are typically two primary ways to acquire this ability: First, by maintaining a supercooled state through the synthesis and accumulation of cryoprotectants. And second, by expanding the supercooling capacity without accumulating cryoprotectants (Storey et al., 1991; Bemani et al., 2012; Heydari and Izadi, 2014; Sinclair et al., 2015; Vrba et al., 2017; Pourani et al., 2019).

Supercooling is a phenomenon that lowers the freezing point of a liquid. The freezing point of a supercooled liquid is known as the supercooling point (SCP). To comprehend the cold tolerance strategy, two pieces of information are essential: the supercooling point and the lower lethal temperature. Based on these two, the insect cold tolerance strategy can be classified into three primary categories:

1) Freeze-intolerant or freeze-avoidant insects: These insects experience high mortality near the SCP. They can tolerate severe winter temperatures by achieving a supercooled state and lowering the SCP. However, they cannot survive if ice forms within their body fluids.
2) Freeze-tolerant insects: These insects can survive extracellular fluid freezing. Mortality primarily occurs at temperatures below the SCP. There is no correlation between the SCP and winter temperatures for these insects.
3) Chill-intolerant insects: Mortality primarily occurs at temperatures significantly higher than the SCP. There is also no correlation between the SCP and winter temperatures for these insects (Rozsypal, 2015; Sinclair et al., 2015).

Enhancing the supercooling capacity involves accumulating cryoprotectants, eliminating ice nucleating agents, and synthesizing hemolymph antifreeze proteins. Additionally, depressing the SCP is closely linked to the cold hardiness of insects. Cryoprotectants, such as low-molecular-weight carbohydrates and polyols, are molecules that lower the melting point of water when dissolved in the extracellular fluid, thus preventing ice formation (Lee et al., 1996; Kar et al., 2018).

The critical thermal minimum (CT_min_) is the temperature at which muscles lose their coordinated function (Terblanche et al., 2007). The disruption of neuromuscular activities is primarily caused by imbalances in ion and water levels, leading to a state known as chill coma. Cold acclimation, through physiological regulations, can effectively mitigate the harmful impacts of cold stress (Enriquez and Colinet, 2019).

The lower lethal temperature (LLT) is the temperature at which a certain percentage of the population dies after a given exposure time. Consequently, LLT_50_ represents the temperature at which 50% of individuals in a population perish following a specific period of exposure. It is important to note that acclimation is generally associated with LLT (Bouchard et al., 2006).

In this study, we investigated the correlation between the SCP, cold tolerance, accumulation of cryoprotectants, and lower lethal temperatures to comprehend the cold tolerance strategy of *H. variegata*, a formidable natural predator. Our findings hold great importance for forecasting the initial population of this promising natural predator early in the season and its possible application in biological pest management programs targeting the common pistachio psylla.

## 2 Materials and methods

### 2.1 Collecting the ladybird beetles

*Hippodamia variegata* adults were collected from pistachio orchards in Kerman, Kerman province, Iran (30° 16′59.56″N, 57° 04′43.64″E) during 2021–2022. They were then placed in 15 × 15 × 15 cm plastic breeding containers with a 5 cm opening diameter on the lid, which was covered with lace for proper ventilation. These containers were used for conducting tests. To investigate the changes in cold tolerance, the supercooling point (SCP), and biochemical characteristics throughout the year, adult insects were collected monthly during the first 6 months (usually in the middle of each month and 24–48 h before the experiment) from the gardens. They were then kept in special containers in the natural environment. Due to the absence of ladybirds in the second half of the year, many insects were collected in September, before the air temperature cooled down. These insects were kept in special containers under natural environmental conditions.

### 2.2 Supercooling points assay

To estimate the SCP, adults were affixed with a thermocouple (NiCrNi probe) using adhesive tape and placed inside a programmable refrigerated chamber. The chamber’s temperature decreased at a rate of 0.5°C per minute. The thermocouple was connected to a Testo 177-T4 automatic temperature recorder (Testo, Germany), enabling temperature readings every 30 s. The data was then extracted using Comsoft 3 Software. The SCP represents the lowest temperature recorded before the release of latent heat resulting from crystallization. In other words, it is the temperature just before a sudden increase in temperature occurs due to the release of internal heat from the insect’s body (Mohammadzadeh and Izadi, 2016). The experiment was conducted for 12 months, with three replications per month, and each replication included five adult insects.

### 2.3 Cold tolerance assay

To assess cold tolerance, we placed adult insects (five replications, each consisting of 12 insects) in a programmable refrigerated chamber. The temperature was gradually reduced from 25°C to the desired temperatures (−10°C to 0°C) at a rate of 0.5°C per minute. The insects were kept at each temperature for 24 h. Subsequently, the temperature was slowly raised (0.5°C per minute) back to 25°C (the optimal growth temperature), and the survival of the insects was assessed after 24 h. Insects that exhibited no movement in their appendages were classified as deceased (Pourani et al., 2019). The experiment was conducted for 12 months, with three replications per month, and each replication included five adult insects.

### 2.4 Low-molecular-weight carbohydrates and polyols assay

Carbohydrates and low-molecular-weight polyols, such as glucose, trehalose, and glycerol, were quantified under laboratory conditions using High-Performance Liquid Chromatography (HPLC). To do this, five insects were carefully cleaned with a brush and weighed. Subsequently, they were homogenized in 1.5–2 mL of 80% ethanol and centrifuged for 15 min at 12000 rpm. The resulting supernatant was evaporated in an oven at 40°C and then reconstituted in 1 mL of HPLC-grade water. Acetonitrile: water (70:30) was employed as the eluent, with a flow rate of 1 mL.min-1. The separation process was conducted at a temperature of 40°C ± 1°C. Standard curves were generated using glucose, trehalose, and glycerol (Heydari and Izadi, 2014; Izadi et al., 2019a). The experiment was conducted for 12 months, with three replications per month, and each replication included an individual adult insect.

### 2.5 The effect of cold acclimation on survival

To assess the impact of gradual exposure to cold temperatures on *H. variegata*, adult insects were moved to a programmable refrigerated chamber. The temperature was then lowered from 25°C to the desired levels (ranging from 0 to −10°C) at a rate of 0.5°C per minute. The insects were kept at each temperature for 24 h before being slowly raised back to 25°C at the same rate of 0.5°C per minute. The survival of the adult insects was observed to determine several key parameters: CT_min_ (the minimum temperature at which insect mortality begins), LT_10_ (the temperature at which 10% of the adults died), LT_50_ (the temperature at which 50% of the adults died), and LT_80_ (the temperature at which 80% of the adults died), all within a 24-h timeframe (Izadi et al., 2019b). The experiment was conducted for 12 months, with three replications per month, and each replication included 12 adult insects.

### 2.6 Induction of chill coma and measurement of chill coma recovery time (CCRT)

Six adult insects were placed in separate clean Petri dishes and transferred to a programmable refrigerated chamber. The chamber was set to temperatures of 2, 1, 0, −1, and −2°C for two and 4 hours. Afterward, the Petri dishes were returned to room temperature (25°C ± 1°C), and the insects were flipped onto their backs. The time it took for the first movement of an appendage (usually a leg or palpus) to be observed was recorded. To account for the possibility of ladybirds pretending to be dead, individuals were gently stimulated with an entomological brush every 5 seconds (Knapp et al., 2018). The experiment was conducted at five temperatures, with five replications per temperature, and each replication included six adult insects.

### 2.7 Statistical analysis

Before analyzing the data, normality was checked using the Kolmogorov-Smirnov test. The test indicates a normal distribution in the dataset. The statistical analyses comparing the means were conducted based on a completely randomized design, employing one-way ANOVA followed by a *post hoc* LSD test at a significance level of α = 0.05 (SPSS 26.0 statistical software). A Student’s t-test was employed to compare the means of two distinct groups (in the analysis of chill-coma recovery time).

## 3 Results

### 3.1 Overwintering strategy

**FIGURE 1:**
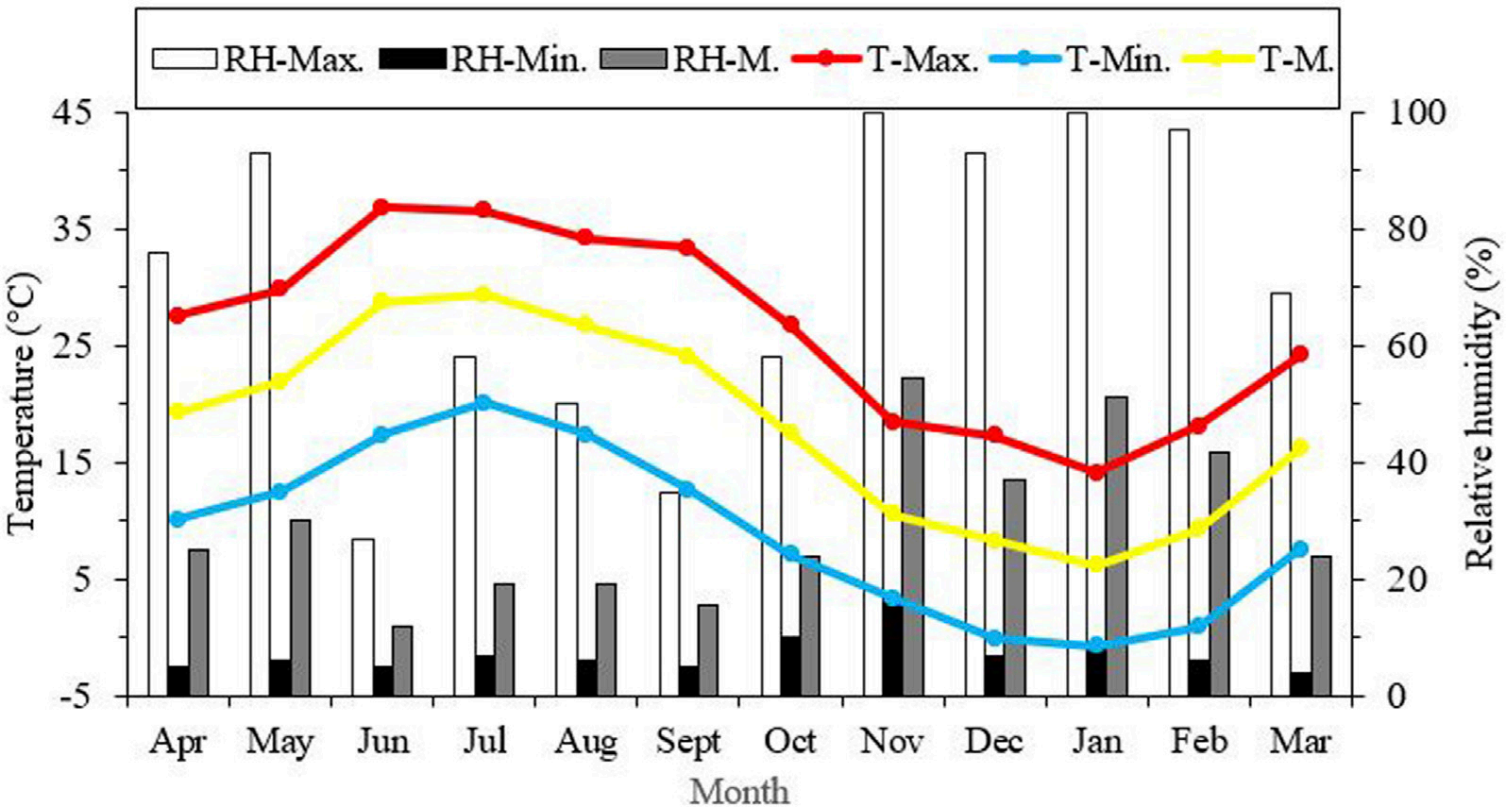
Seasonal variations in average, lowest, and highest ambient temperature and relative humidity in Kerman from April 2021 to March 2022. T = Temperature, RH = relative humidity, Max = Maximum, Min = Minimum, M = Mean.

Seasonal variations in the average, minimum, and maximum ambient temperatures in Kerman from April 2021 to March 2022, as well as changes in the supercooling point (SCP) during the overwintering period of *H. variegata*, are depicted in Figures 1, 3, respectively. During the non-overwintering period, which spans from late April to October, the mean SCP of adult beetles was around −13.5°C. However, starting from October, there was a significant decrease in SCP (df = 11, F_11,24_ = 4576.43, *P* < 0.001), reaching its lowest point of −23.2°C in February. From March onwards, the SCP started to rise again, reaching the same level as the active phase of the beetle’s lifespan. By examining Figures 1, 3 together, it can be inferred that the lowest SCP coincides with the lowest ambient temperature.

**FIGURE 2:**
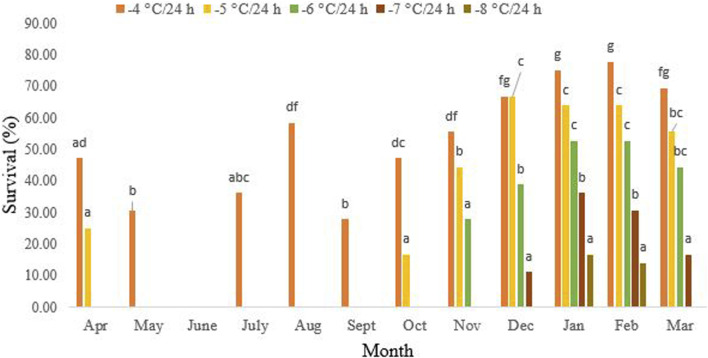
Change in the cold tolerance of *Hippodamia variegate* during the overwintering period. To assess cold tolerance, we exposed adult beetles to various subzero temperatures for 24 h. The greatest cold tolerance was observed in February. Different letters in columns of the same color indicate a significant difference (*p* = 0.05, LSD test).

Figure 2 presents data demonstrating that non-overwintering adults were unable to withstand exposure to −5 °C/24 h. During this period, the highest survival rate of 58.3% was recorded after 24-h exposure to −4°C in August (df = 11, F_11,24_ = 21.31, *P* < 0.001). However, starting in October, their cold tolerance significantly increased. By December to February of the following year, the highest level of tolerance, at 66.3%, was observed, where 16.3% of the beetles were able to endure −5°C for 24 h. Interestingly, in October (df = 11, F_4,10_ = 8.94, *p* = 0.002) and November (df = 11, F_3,8_ = 7.86, *p* = 0.009), the beetles exhibited tolerance to −6°C and −7°C/24 h, respectively. In January, the cold tolerance of the overwintering beetles reached its peak at 16.6% after a 24-h exposure to −8°C. However, from March onwards, the cold tolerance decreased again (df = 11, F_1,4_ = 0.25, *p* = 0.64). No survival was recorded in March following a 24-h exposure to −8°C.

### 3.2 Changes in low-molecular-weight (LMW) carbohydrate and polyol contents

Figure 4 illustrates the changes in the concentration of two low molecular weight (LMW) carbohydrates, namely, glucose and trehalose, as well as a polyol called glycerol. The concentrations of trehalose (∼2.0 mg/g), glycerol (∼2.0 mg/g), and glucose (∼1.0 mg/g) were at their lowest levels from April to July. Subsequently, they reached their peak levels (11.15, 10.82, and 6.31 mg/g fresh body weight, respectively) in September, and then gradually declined to the same levels observed in July. Notably, the concentrations of trehalose and glycerol were consistently higher than that of glucose (Glucose, df = 11, F_11,24_ = 9.85, *P* < 0.001; Trehalose, df = 11, F_11,24_ = 23.08; *P* < 0.001; Glycerol, df = 11, F_11,24_ = 21.85, *P* < 0.001).

### 3.3 Low lethal temperatures

The critical thermal minimum (CT_min_) reached its highest level of approximately 0.4°C in July, while its lowest level was recorded at −2.2°C in January and February (Figure 5) (df = 11, F_11,24_ = 3.05, *p* = 0.11). Notably, the highest and lowest values for LLTs coincided with the hottest and coldest months of the year, respectively. Additionally, the lowest point of LLT coincided with the lowest level of the SCP and the highest level of cold tolerance. As the ambient temperature and the SCP decreased, so did the lower lethal temperature. The highest LT_50_ was observed from July to September, aligning with the maximum ambient temperature, the highest SCP, and the lowest cold tolerance. From September onwards, LT_50_ gradually decreased and reached its lowest point of −5.3°C in January and February (Figure 5) (df = 11, F_11,24_ = 3.96, *p* = 0.002). This point coincided with the lowest level of the SCP and the highest level of cold tolerance. In June, with a minimum ambient temperature of 17.4°C, the lower lethal temperature required to kill 80% (LLT_80_) of the population was −3.0°C. In contrast, this value decreased to its lowest point of −7.9°C in February, with a minimum ambient temperature of 0.9°C (df = 11, F_11,24_ = 9.22, *P* < 0.001).

### 3.4 Chill-coma recovery time (CCRT)

The results in Figure 6 show that the CCRT increased as the exposure temperature and time decreased. When the beetles were exposed to different temperatures for 2 hours, the CCRT levels varied. The lowest level recorded was 17.2 s at 2°C (df = 4, t = −4.45, *p* = 0.002), while the highest level reached was 68.0 s at −2°C (df = 4, t = 10.64, *p* = 0.002). By increasing the exposure time to 4 h, the lowest and highest levels of the CCRT were 21.7 s (df = 4, t = 4.45, *p* = 0.002) and 101.7 s (df = 4, t = 10.64, *p* = 0.002), respectively.

## 4 Discussion

**FIGURE 3:**
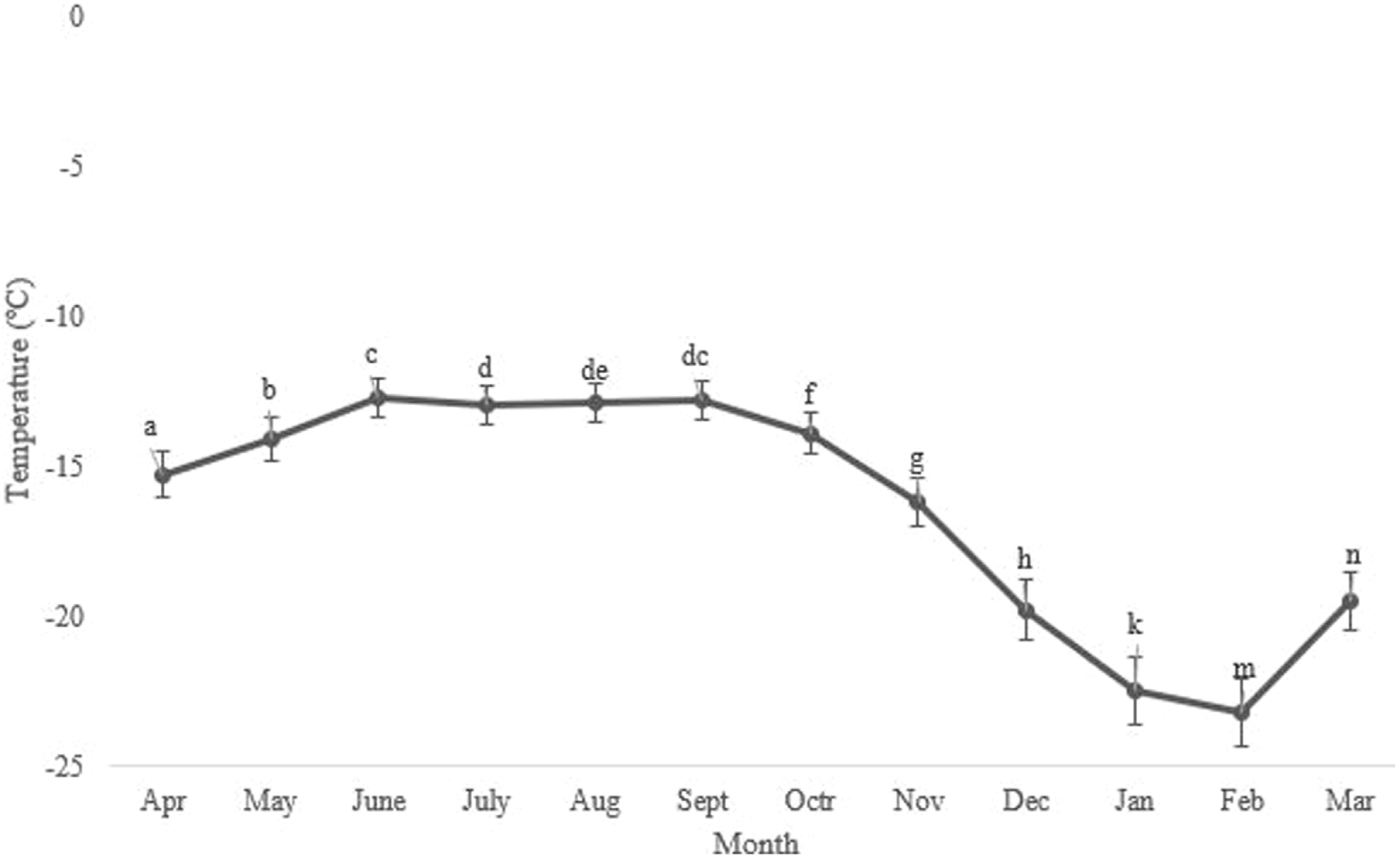
Change in the supercooling point (SCP) of *Hippodamia variegata* during the overwintering period. The lowest SCP was observed in February. Different letters indicate a significant difference (*p* = 0.05. LSD test).

From late April until October, pistachio trees are always active and harbor different pests, especially the common pistachio psylla. By harvesting the nuts in late September and early October, after defoliation, the pistachio trees enter a period of winter dormancy that lasts through February (Rahemi and Pakkish, 2009). By taking into account the biological characteristics of pistachio trees, fluctuations in ambient temperature, and the SCP, we can infer that the peak supercooling point (SCP) of the beetles aligns with the highest ambient temperature during the trees’ active period when they serve as hosts for the pests. By decreasing the temperature, the trees undergo defoliation and enter dormancy. These changes occur simultaneously with the overwintering of the common pistachio psylla and its predator, *H. variegata*. So, there has been a continuous decrease in the SCP of the predators since October, which was in line with the drop in environmental temperature. Eventually, the lowest level of the SCP was achieved in January and February, when the ambient temperature reached its minimum (Figures 1, 3). Commonly, from late February onward, the environmental temperature gradually rises, and pistachio trees end their dormancy. This period is accompanied by the invasion of the common pistachio psylla and, subsequently, their predators, such as *H. variegata*. By the end of the beetle’s overwintering period, their SCP had steadily increased and reached the same level as April. Taking these facts into consideration, we would like to conclude that naturally occurring cold acclimation during autumn is a preparatory phenomenon that leads to a gradual enhancement of the supercooling point (SCP) and cold tolerance of the beetles. In agreement with this conclusion, a previous study (Hamedi and Moharramipour, 2013) demonstrated that prolonged exposure of adult beetles to subzero temperatures is necessary for the successful completion of the *H. variegata* overwintering process. The supercooling point of the seven-spot ladybird, *Coccinella septempunctata* L., exhibited a similar pattern to that of *H. variegata*. It decreased during the overwintering period and reached its lowest level of approximately −21°C in December and January (Nedved, 1993). The supercooling point of the overwintering multicolored Asian ladybird, *Harmonia axyridis* (Pallas), in Europe, ranged from −17.5 to −16.5°C (Berkvens et al., 2010). In comparison, the SCP of this biological control agent in America (Minnesota and Georgia) decreased significantly during the winter months and reached its lowest level of approximately −23.0°C in February (Koch et al., 2009). The first study (Berkvens et al., 2010) revealed that the supercooling point of the indoor overwintering population of *H. axyridis* increased as winter progressed and reached its highest level at −13.2°C. This finding also explains the impact of natural acclimation on the reduction of SCP and the improvement of cold tolerance. The supercooling capacity of the firebug, *Pyrrhocoris apterus* (Hemiptera: Pyrrhocoridae) is closely linked to climatic conditions, which validates the use of SCP as a suitable measure of cold tolerance in this species. The most significant relationship was observed between the average SCP and the lowest winter temperature. Furthermore, the number of cold days was linked to the average population SCP, as well as the number of freezing days (Ditrich et al., 2018). In the Mediterranean fruit fly, *Ceratitis capitata*, the SCP of various populations was determined by their geographical distribution (Papadopoulos et al., 2023). Another study revealed that long-term acclimation significantly enhanced the SCP of the seed beetle, *Megabruchidius dorsalis* (F.) (Col.: Chrysomelidae: Bruchinae) (Chen et al., 2023).

In our study, from October onwards, the reduction of the SCP was accompanied by an enhancement in cold tolerance (Figures 2, 3). The highest cold tolerance was observed in February, with the lowest SCP and the lowest ambient temperature. Before the onset of overwintering, from May to September, the adult beetles can only survive temperatures as low as −4°C/24 h, with the highest survival rate occurring in August. No survival was recorded at −5°C for 24 h during these months. From October onward, the cold tolerance increased, and the beetles were partially able to tolerate −5°C for 24 h. From November to January, this partial cold tolerance increased to −6°C/24 h and −7°C/24 h, respectively. Only, in January and February, did a small percentage of the population tolerate −8°C for 24 h. However, no survival was recorded for temperatures lower than this, even in January and February, when the supercooling point was at its lowest and cold tolerance was at its highest. So, the cold tolerance of this ladybird beetle is far above the SCP. This convinced us to classify this species as a chill-susceptible insect. However, the ability of chill-susceptible insects to withstand cold temperatures is determined by their capacity to survive exposure to temperatures above their supercooling point (SCP). Nevertheless, some researchers categorize this cold tolerance strategy into two groups: chill-susceptible (perishing due to mild chilling) and chill-tolerant insects (perishing due to severe chilling above the SCP) (Overgaard and MacMillan, 2016).

We also conclude that a well-developed SCP is most likely responsible for the survival of the overwintering adults of *H. variegata*. That means the adults enhanced their supercooling capacity by significantly reducing the SCP during the winter, when temperatures dropped below zero, to adapt to this challenging environmental condition. Freeze-avoiding animals typically depend on a well-developed supercooling point to prevent the formation of ice in their extracellular fluid at temperatures below freezing (Sømme et al., 1982; Sinclair, 1999; Storey and Storey, 2017; Toxopeus and Sinclair, 2018). This is a common occurrence in overwintering adults of *H. axyridis* (Watanabe, 2002). Animals employing this cold tolerance strategy typically use various mechanisms to prevent the freezing of their extracellular fluids. These mechanisms often involve emptying their digestive tract and producing and storing cryoprotectants, which help increase their ability to supercool (Lee et al., 1989; Bale, 1996). Moreover, suppose the difference between supercooling point (SCP) and lower lethal temperature (LLT) is significant (as in this study, where the lowest SCP was −23.2°C whereas the LT_50_ was −5.3°C). In that case, the lower lethal temperature may also serve as a reliable indicator for determining the cold tolerance strategy. In such a way that LT_50_ is larger than SCP, the insect is chill susceptible (LT_50_ > SCP = chill susceptible) (Sinclair et al., 2015). Our previous study (Pourani et al., 2019) showed that in *Cheilomenes sexmaculata* (F.) and *Oenopia conglobata contaminata* (Menetries) (Col.: Coccinellidae), the highest mortality occurred above the SCP. So, these ladybird beetles are also considered chill-intolerant species.

**FIGURE 4:**
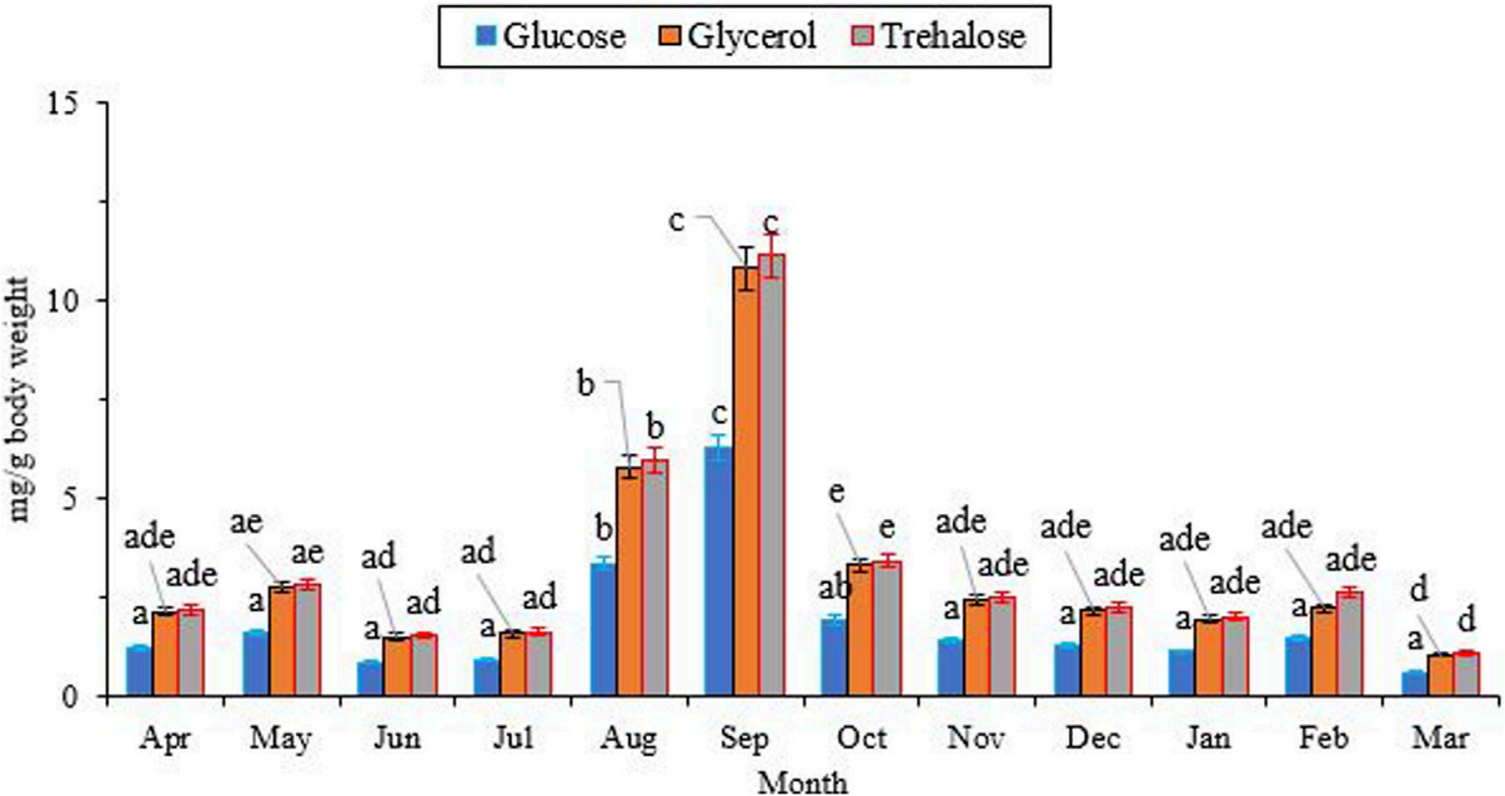
Change in the low molecular weight carbohydrates of *Hippodamia variegata* during the overwintering period. The highest level of the cryoprotectants was observed in Sep. Different letters on each line indicate a significant difference (P1.05, LSD test).

Changes in the concentration of low molecular weight (LMW) carbohydrates and polyols followed the same trend, with the highest level at the beginning of the overwintering period and the lowest level in the well-developed overwintering adults (Figure 4). Interestingly, the changes in the concentration of these potential cryoprotectants were opposite to what was expected. The synthesis and accumulation of cryoprotectants typically coincide with the expansion of the supercooling point and the improvement of cold tolerance in the majority of freeze-intolerant insects (Watanabe, 2002; Bemani et al., 2012; Heydari and Izadi, 2014; Hasanvand et al., 2020). With this assumption, we expected the highest levels of these components in January and February, along with the lowest supercooling point and the highest cold tolerance. However, unexpectedly, we encountered the highest level of these compounds at the beginning of overwintering. This finding convinces us to conclude that this species primarily relies on expanding the supercooling point to enhance its cold tolerance. We also conclude that this species does not utilize the three sampled carbohydrates (glucose, trehalose, and glycerol) as cryoprotectants. Conversely, in a different study, overwintering adults of *H. variegata* showed a 45-fold increase in myo-inositol and a five-fold increase in glucose content when exposed to subzero temperatures, suggesting that myo-inositol and glucose are the primary cryoprotectants for this species (Hamedi et al., 2013). Comparing these two studies, conducted in different areas with substantial geographical and environmental variations, indicates the influence of geographical distribution and population disparities on the cold tolerance strategy of this predatory ladybeetle. The cold tolerance strategy of insects is influenced by several factors, including geographical distribution and population variations. For instance, a study on the cold tolerance of the seed beetle, *M*. *dorsalis* found that the species’ cold tolerance strategy varied under different temperature and time stresses (Chen et al., 2023). Other studies also found that the cold tolerance of insects is linked to the regional climate (Yoshio and Ishii, 1998; Zhou et al., 2011; Xie et al., 2015; Weldon et al., 2018). However, our findings highlight the role of SCP depression, as a viable cold-tolerance strategy in adult *H. variegata*. On the other hand, the significant increase in the levels of these compounds starting in July could indicate the beginning of overwintering, which coincides with a significant drop in the surrounding temperature. The decrease in ambient temperature may act as a stressor, stimulating the accumulation of potential cryoprotectants to enhance the cold tolerance of beetles when the SCP has not yet been depressed. By decreasing the SCP from October onward, the supercooling capacity is enhanced by shifting the cold tolerance strategy from cryoprotectant accumulation to a well-expanded SCP. So, cryoprotectants are used as a source of fuel during the first months of overwintering, leading to a significant decrease in their levels from September onward.

**FIGURE 5:**
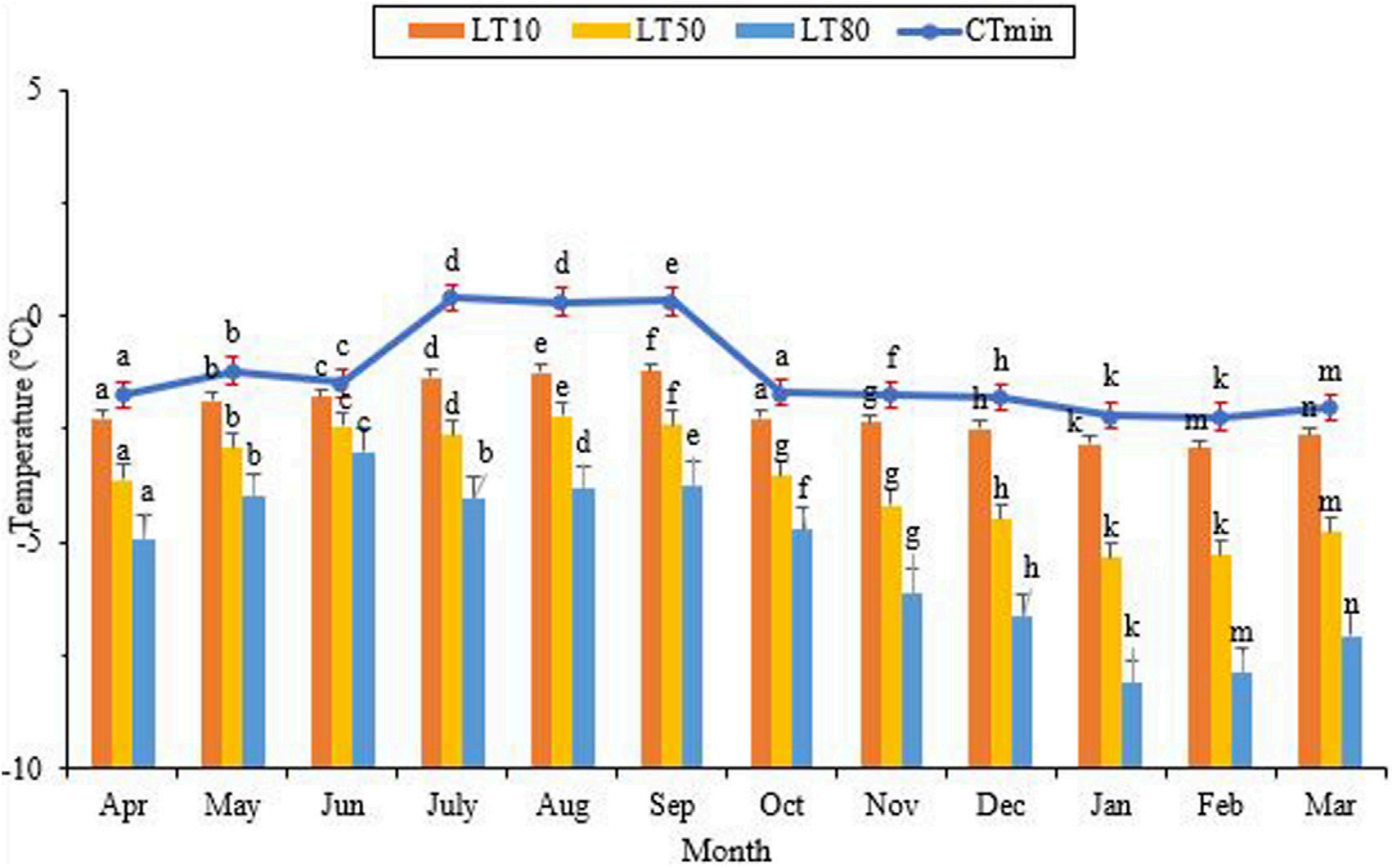
Change in the lower critical temperatures of *Hippodamia variegata* during the ovenvintering period. Different letters on columns with the same color indicate a significant difference (*p* = 0.05, LSD test).

Starting in September, there was a consistent decline in critical temperature minimum (CT_min_) and lower lethal temperature (LLT) as the overwintering period progressed. The lowest critical and lethal temperatures were recorded when the ambient temperature and the SCP were at their minimum levels, while the cold tolerance was at its maximum level (Figures 2, 5). This finding also confirms the direct relationship between natural cold acclimation and the expansion of the lethal temperature range. In temperate zones, insects typically use seasonal thermal fluctuations as a cue to enhance their ability to withstand cold temperatures (Andersen et al., 2016). Enhancing cold tolerance is a physiological process that involves the maintenance of a transmembrane ion current gradient and the subsequent preservation of the cell membrane potential (Andersen et al., 2016). In this study, the researchers found that cold acclimation improved the cold tolerance of adult migratory locusts, *Locusta migratoria* (Orth. Acrididae). This improvement was attributed to the maintenance of ion homeostasis and membrane potential. Chill injury is commonly associated with the disruption of ion homeostasis, which results in impairing the levels of potassium (K^+^) and calcium (Ca^2+^) in the hemolymph. This, in turn, causes depolarization of the cell membrane and ultimately leads to cell death (Andersen et al., 2016). Cold acclimation improved the cold tolerance and metabolic stability of *Drosophila melanogaster* (Koštál et al., 2011; Enriquez et al., 2018; Enriquez and Colinet, 2019). Our previous study also confirmed that cold acclimation significantly improved the cold tolerance of *Trogoderma granarium* Everts (Col.: Dermestidae) larvae (Mohammadzadeh and Izadi, 2018).

**FIGURE 6:**
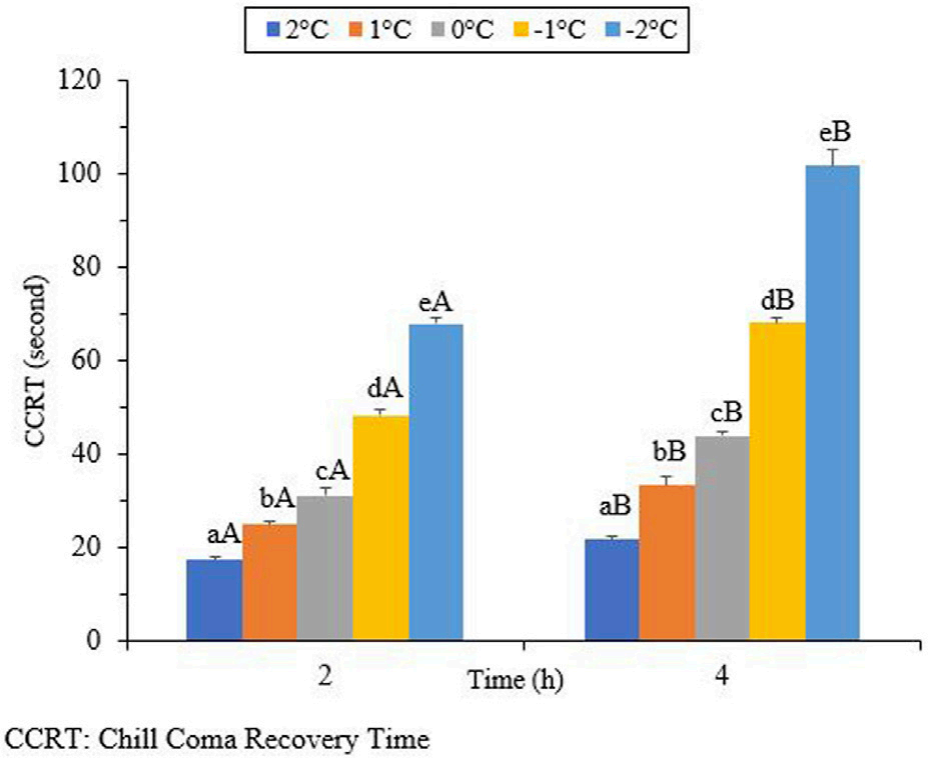
Chill coma recovery time (CCRT) of *Hippodamia* *variegata* (different lowercase letters indicate a significant difference among the temperatures (LSD), and different uppercase letters indicate a significant difference between the exposure times (Student’s t-test) (P-41.05).

Chill coma refers to a temporary and reversible physiological state that is triggered by cold exposure. It is characterized by the cessation of both electrophysiological signals and muscle activity (Hazell and Bale, 2011; Davis et al., 2021). Chill-coma recovery time (CCRT) is an indicator of low, but non-lethal, thermal tolerance (Ransberry et al., 2011; Gerken et al., 2016). Chill-coma recovery time can also be used as a reliable predictor of cold tolerance in certain insects. However, in the current study, changes in the CCRT were found to be associated with exposure time and temperature (Figure 6). The chill coma recovery time, which measures the duration for insects to recover from a comatose state, is known to reflect ecologically relevant variations associated with distribution, adaptation, and/or acclimation (Findsen et al., 2014). Research on the Mediterranean fruit fly, *Ceratitis capitata* (Diptera: Tephritidae), has shown that chill coma recovery time is affected by both latitude and macroclimatic conditions (Moraiti et al., 2022). Our results indicate the negative impacts of ambient temperature and exposure time on CCRT. By decreasing the temperature and increasing the exposure time, the CCRT decreased. The highest CCRT was observed when the beetles were exposed to −2°C for 4 hours. We can conclude that the CCRT is not a reliable predictor of cold tolerance in overwintering adults of *H. Variegata* because this trait is not directly linked to their survival during the winter. The same results were presented for the cold tolerance strategy of *H. axyridis* (Knapp et al., 2018).

## 5 Conclusion

The overwintering adults of *H. variegata* do not use the three sampled carbohydrates (glucose, trehalose, and glycerol) as cryoprotectants but may utilize other carbohydrates for this purpose. Moreover, when comparing the supercooling point (SCP) and cold tolerance of the beetles, it is evident that the majority of the mortality occurs significantly above the SCP. Therefore, it can be concluded that the overwintering adults of *H. variegata* are chill-susceptible insects with the ability to significantly lower their supercooling point to survive the low winter temperatures. Given this species’ ability to survive long periods of sub-zero temperatures above the SCP, it is not inaccurate to classify this insect as a chill-tolerant species as well. The supercooling point is commonly used as a reliable metric for evaluating the cold tolerance of insects. In freeze-intolerant species, this metric indicates the lower lethal temperature (LLT) (Andreadis and Athanassiou, 2017; Ditrich, 2018; Li et al., 2021). However, the SCP and LLT could be used as reliable predictors of the cold hardiness of this ladybird beetle. Moreover, the subzero temperature is expected to be the main limiting factor for surviving the overwintering period. In line with this finding, research on the South American tomato pinworm, *Tuta absoluta*, comparing the Lower lethal temperature (LLT) with January temperatures revealed that extremely low temperatures could kill more than 90% of the overwintering pest population (Li et al., 2021).

## Data Availability

The original contributions presented in the study are included in the article/Supplementary materials, further inquiries can be directed to the corresponding author.
